# Validation of microinjection methods for generating knockout mice by CRISPR/Cas-mediated genome engineering

**DOI:** 10.1038/srep04513

**Published:** 2014-03-28

**Authors:** Takuro Horii, Yuji Arai, Miho Yamazaki, Sumiyo Morita, Mika Kimura, Masahiro Itoh, Yumiko Abe, Izuho Hatada

**Affiliations:** 1 Laboratory of Genome Science, Biosignal Genome Resource Center, Institute for Molecular and Cellular Regulation, Gunma University, 3-39-15 Showa-machi, Maebashi, Gunma 371-8512, Japan; 2 Division of Developmental Biotechnology, Department of Bioscience and Genetics Research Institute, National Cerebral and Cardiovascular Center, 5-7-1 Fujishiro-dai, Suita Osaka 565-8565, Japan; 3 Department of Laboratory Sciences, Graduate School of Health Sciences, Gunma University, 3-39-22 Showa-machi, Maebashi, Gunma 371-8514, Japan; 4 Department of Obstetrics and Gynecology, Gunma CHUO General Hospital, 1-7-13, Kouun-cho, Maebashi, Gunma 371-0025, Japan; 5 These authors contributed equally to this work.

## Abstract

The CRISPR/Cas system, in which the Cas9 endonuclease and a guide RNA complementary to the target are sufficient for RNA-guided cleavage of the target DNA, is a powerful new approach recently developed for targeted gene disruption in various animal models. However, there is little verification of microinjection methods for generating knockout mice using this approach. Here, we report the verification of microinjection methods of the CRISPR/Cas system. We compared three methods for injection: (1) injection of DNA into the pronucleus, (2) injection of RNA into the pronucleus, and (3) injection of RNA into the cytoplasm. We found that injection of RNA into the cytoplasm was the most efficient method in terms of the numbers of viable blastocyst stage embryos and full-term pups generated. This method also showed the best overall knockout efficiency.

Mouse is the most widely used mammalian model organism. However, conventional gene targeting methods using homologous recombination in mouse embryonic stem (ES) cells are generally used for genetic research. The recent development of site-specific endonucleases for selective genome cleavage has been an important advancement in genome engineering. These enzymes include zinc-finger nucleases (ZFN)^1^ and transcription activator-like effector nucleases (TALEN)^2^. ZFN and TALEN are composed of programmable, sequence-specific DNA-binding modules linked to a non-specific DNA cleavage domain. Although these technologies are widely used in animals other than mouse, they have not been used much in mouse, principally because ZFN and TALEN are labor intensive and expensive techniques that do not have substantially better performance compared to ordinary gene knockout technology. More recently, an epoch-making technology using clustered regularly interspaced short palindromic repeats (CRISPR) and RNA-guided Cas9 nucleases^3^ has been developed and applied to gene disruption in mammals^4^,^5^. CRISPR RNA-guided Cas9 nucleases use small base-pairing guide RNAs (gRNAs) to target and cleave foreign DNA elements in a sequence-specific manner^6^. Many researchers have taken note of this technology because it is easy and quick. Furthermore, it can be used to generate mice carrying mutations in multiple genes in one step^7^, which was not possible with previous methods. Wang et al. reported these multiple mutant mice by cytoplasmic microinjection of RNAs encoding the Cas9 nuclease and gRNAs.

However, this study left a number of issues unresolved. For example, which microinjection method is the most successful for generating knockout mice. Two components are introduced into oocytes to make knockout mice by the CRISPR system. One is the Cas9 nuclease RNA and the other is a gRNA complementary to the target DNA. The most direct method would be to inject the vectors for Cas9 and gRNA into the pronucleus; however, this method has the risk of integration of the vectors into the chromosomes, even when using circular plasmids^8^. Therefore, injection of *in vitro* transcribed RNA would be a better alternative. However, Cas9 RNA and the gRNA work in different locations, the cytoplasm and pronucleus, respectively. Theoretically, it would be best to inject each RNA into the cytoplasm or pronucleus, but, in practical terms, such injections are very difficult. Here, we report a comparison of three different injection methods: (1) injection of DNA into the pronucleus, (2) injection of RNA into the pronucleus, and (3) injection of RNA into the cytoplasm. We found that the injection of RNA into the cytoplasm was the most efficient method and yielded the greatest numbers of normal blastocyst stage embryos and full-term pups. This method also showed the best overall knockout efficiency.

## Results

### Design and construction of CRISPR

**Figure 1:**
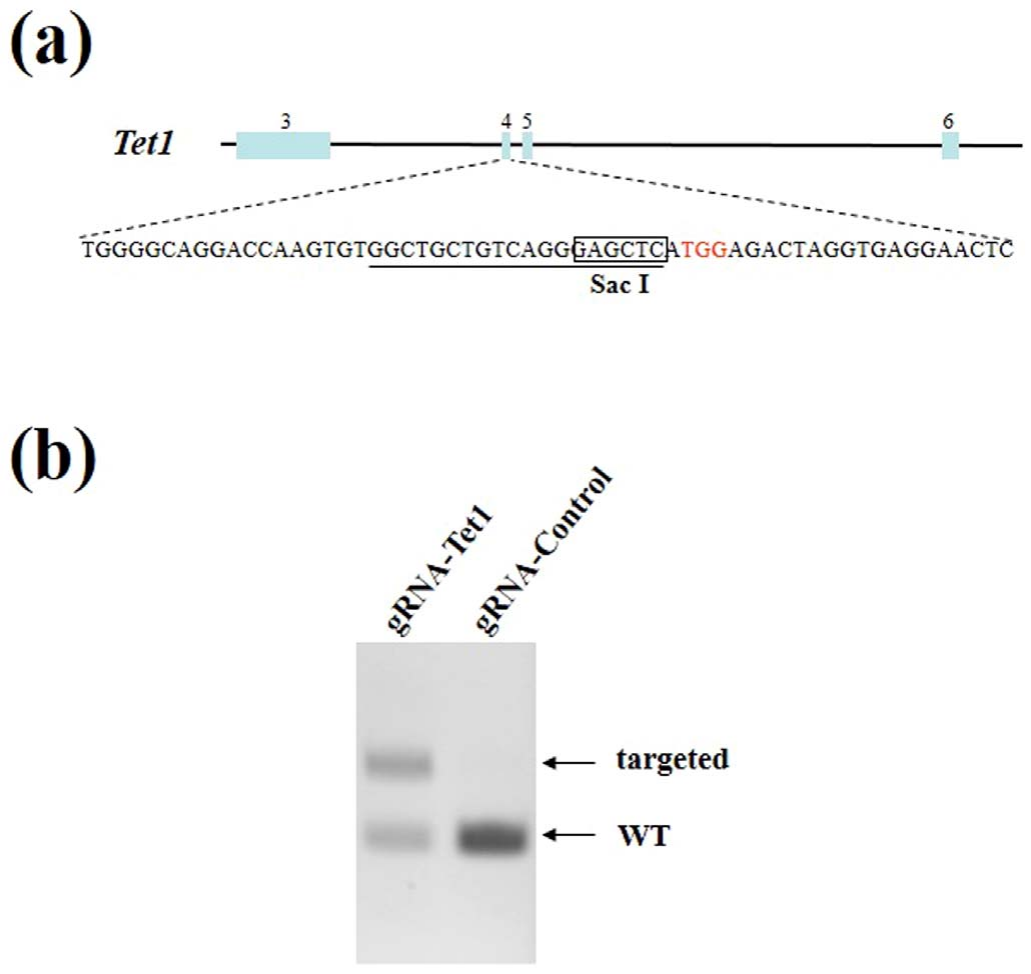
The Cas9/gRNA-targeting site in mouse *Tet1* and validation of its targeting efficiency. (a) The Cas9/gRNA-targeting sites in mouse *Tet1*. The gRNA-targeting sequence is underlined and the PAM sequences are indicated in red. Exons are indicated by closed boxes and the boxed sequence indicates the Sac I restriction site in the target region. (b) Validation of targeting efficiency of the *Tet1* gene using mouse embryonic stem cells. PCR products were digested with the restriction enzyme Sac I that cleaves at the Cas9 endonuclease target site and then analyzed by gel electrophoresis. PCR products generated from DNA containing successfully targeted *Tet1* were uncleaved and, therefore, larger than the product generated from the control DNA. The intensity of each fragment was measured and the targeting efficiency was calculated.

We designed a gRNA for exon 4 of the *Tet1* gene, which encodes a member of the tet methylcytosine dioxygenase family (Figure 1A). Tet proteins convert 5-methylcytosine to 5-hydroxymethylcytosine (5hmC), and this process is an important part of DNA demethylation^9^. The CRISPR target site must perfectly match the PAM sequence (NGG) and the 12 bp seed sequence at the 3′ end of the gRNA^3^. Therefore, a 23-mer sequence (N21GG) from exon 4 of the *Tet1* gene whose 16 bp sequence (N14GG) did not cross-react with any other sites in the mouse genome was selected and used to construct the gRNA expression vector. To examine the knockout efficiency of the gene, ES cells were co-transfected with the Cas9 expression vector and the gRNA vector targeting *Tet1*. The cells were collected 48 h after transfection and DNA was extracted. The targeted locus contains a Sac I restriction site; therefore, successful targeting is indicated by a disruption of the Sac I site. The digested product was subjected to electrophoresis and the knockout efficiency of the *Tet1* gene was determined (Figure 1B). The *Tet1* loci was successful targeted (55%), as indicated by disruption of the Sac I site, while cells transfected with the Cas9 expression vector and a control gRNA vector showed no disruption of the Sac I site. Thus, this gRNA was applicable for making knockout mice.

### Comparison of the effects of different microinjection methods on *in vitro* development to the blastocyst and full-term stages with CRISPR

**Figure 2:**
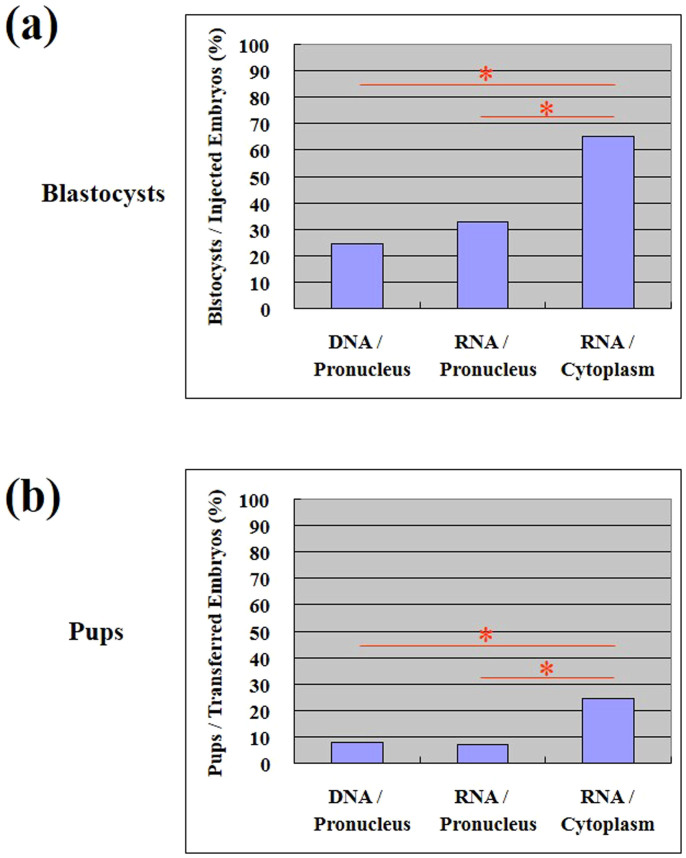
Viability of the *Tet1* targeted mice. (a) Percentage of viable blastocysts per injected embryos. *, P < 0.05. (b) Percentage of pups per transferred embryos. *, P < 0.05.

We compared three methods for injection: (1) injection of DNA into the pronucleus, (2) injection of RNA into the pronucleus, and (3) injection of RNA into the cytoplasm. RNAs for injection were transcribed in vitro and DNAs were circular plasmids. The nucleic acids were injected into the cytoplasm or pronucleus of 1-cell stage C57BL/6 embryos. After injection, the zygotes were immediately transferred into pseudopregnant female mice. The developmental efficiency to the blastocyst stage is expressed as the percent of the total injected embryos developing to the blastocyst stage. At the blastocyst stage, the developmental efficiency for methods (1), (2), and (3) was 24%, 33%, and 65%, respectively (Figure 2a, Table 1). Thus, the developmental of embryos that received injected RNA into the cytoplasm was significantly higher than the other injection methods. The full-term developmental efficiency is expressed as the percent of embryos developing to full term relative to the total transferred. At full term, the developmental efficiency for methods (1), (2), and (3) was 8%, 7%, and 24%, respectively (Figure 2b, Table 2). Again, successful development of zygotes that received an RNA injection into the cytoplasm was significantly greater than the other methods in producing full-term animals. We also designed a gRNA for exon 7 of the *Tet1* gene. Similar results were also obtained for this target (Table 2).

### Comparison of the effects of microinjection methods on the targeting efficiency with CRISPR

**Figure 3:**
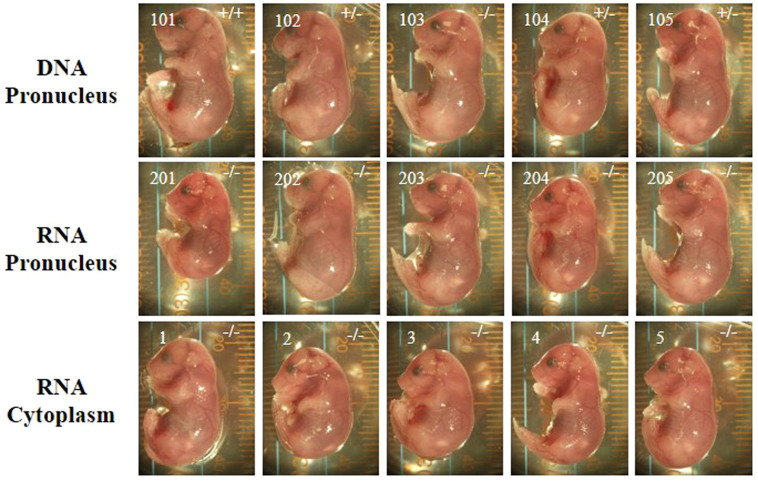
Newborn mice generated by the CRISPR method. Five pups generated by each injection method are shown. *Tet1* genotypes are indicated in each image.

**Figure 4:**
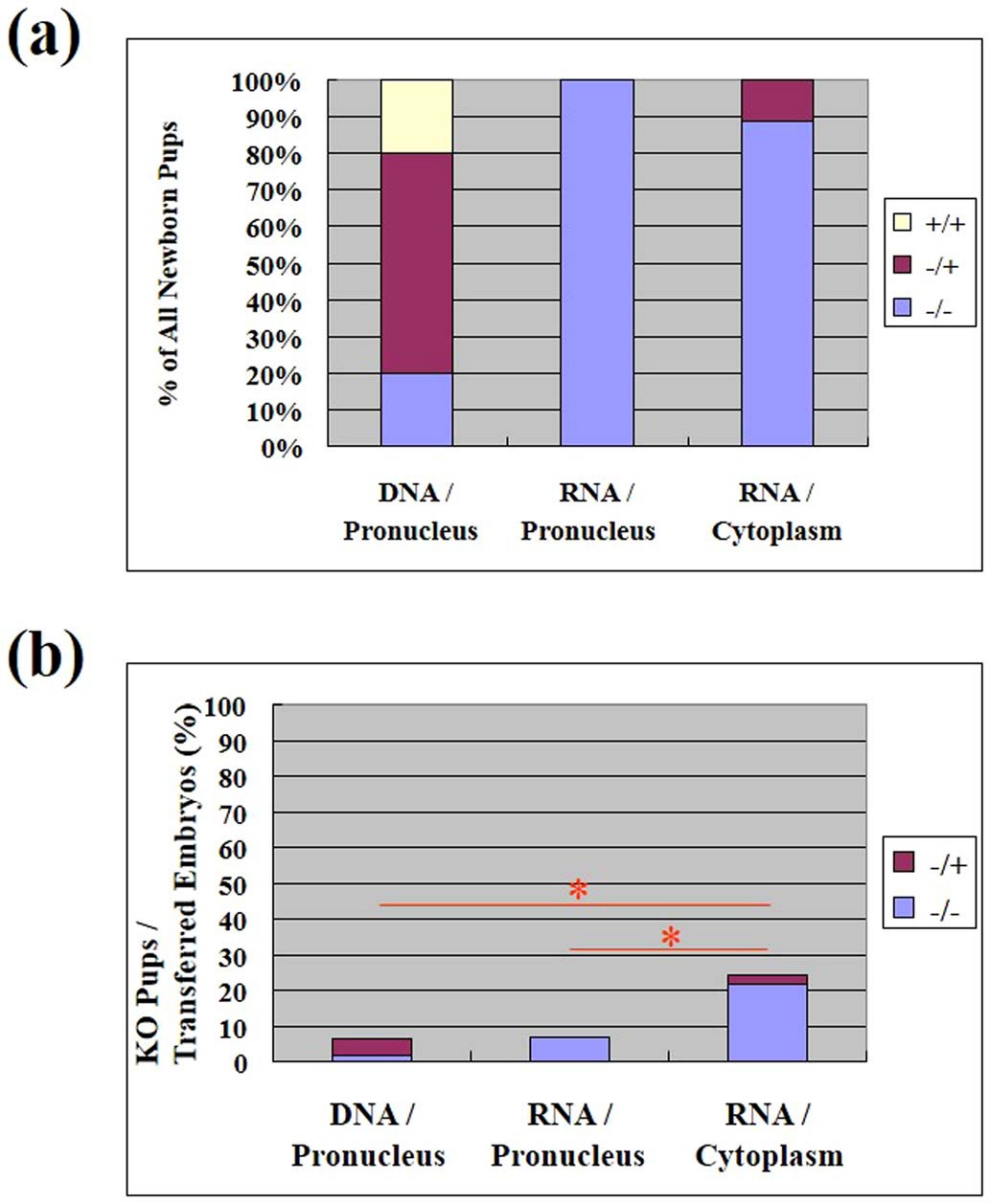
Knockout efficiencies of each injection method. (a) Percent of each *Tet1* genotype among all newborn mice generated by each injection method. (b) Overall efficiencies of generating knockout mice (+/− or −/−) by each of the three injection methods. Numbers of knockout mice per transferred embryos are presented. *, P < 0.05.

Successful targeting was indicated by a disruption of a Sac I restriction site that is located in the predicted cleavage site. Successfully targeted alleles were uncleaved and wild-type alleles underwent complete cleavage. Genomic DNA was extracted from tail snips of the mouse pups, and the sequence spanning the target site of *Tet1* was amplified by PCR. The PCR products were digested with Sac I and subjected to gel electrophoresis for genotyping. There were no apparent phenotypic differences among the knockout mice generated by the three methods (Figure 3). We analyzed body weight of *Tet1* knockout mice and found that the homozygote showed significantly lower birth weight than wild-type mice as reported previously^10^ (Supplementary Figure 1). The genotype of *Tet1* newborn mice showed that percentages of gene disrupted mice per newborn pups were similar among three injection conditions (1) injection of DNA into the pronucleus (80%), (2) injection of RNA into the pronucleus (100%), and (3) injection of RNA into cytoplasm (100%) (Figure 4a, Table 2). However, the percentages of homozygous knockouts per newborn pups were significantly lower in the mice receiving an injection of DNA into the pronucleus, compared to the other two methods. The genotyping of *Tet1* in newborn mice also showed that the mice receiving an injection of RNA into the cytoplasm had a significantly higher percentage of knockouts per transferred embryos than either of the other two methods (Figure 4b, Table 2). For a gRNA for exon 7, similar results were also obtained for this target (Table 2).

### Analysis of mutations generated by CRISPR

**Figure 5:**
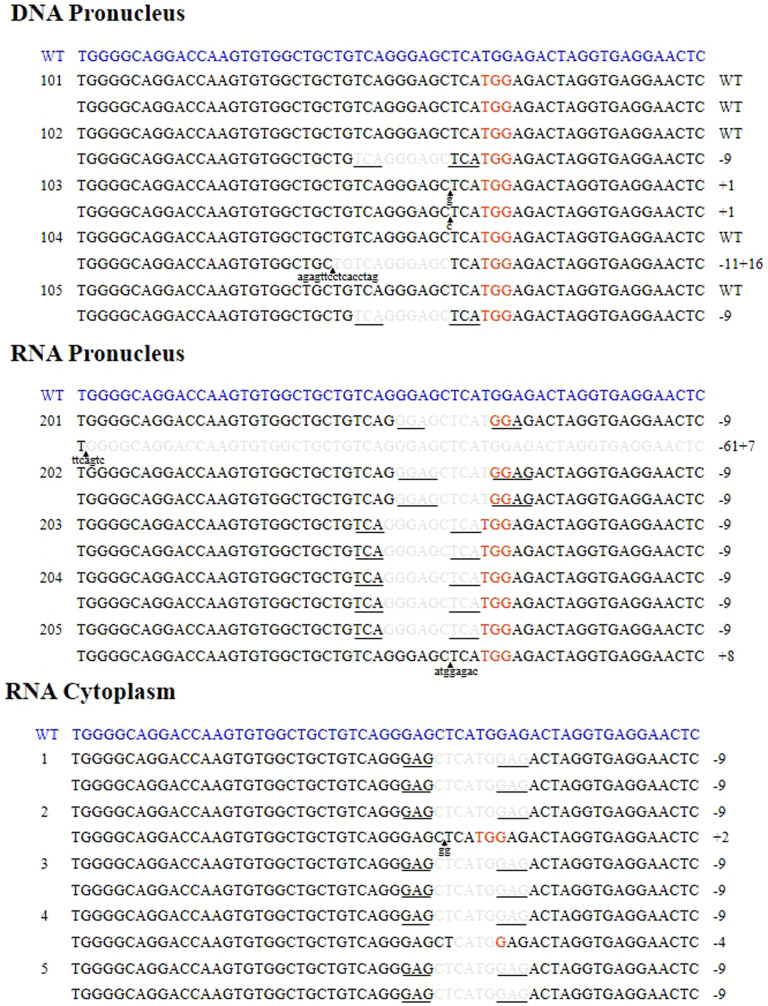
Analysis of mutations generated by CRISPR. Genotyping of newborn mice generated by the CRISPR method. The two *Tet1* alleles are presented for each mouse. The PAM sequences are shown in red. Deletions are indicated in grey letters. Lower case letters indicate insertion mutations and arrows indicate the sites of insertions. Micro-homology flanking the cleavage site is underlined in the sequence.

To determine the mutations generated by CRISPR in individual founders, we analyzed the sequence of the *Tet1* exon 4 from five pups per injection method. PCR products were TA cloned and sequenced. The *Tet1* sequences of the two alleles of each pup are shown in Figure 5. The mutations were deletions and/or insertions around the cutting sites near the PAM (NGG) sequence. The size of the deletions ranged from 4 to 61 bp, with an average length of 11 bp. Interestingly, most of the biallelic mutant mice have the same deletions. All of these mutations are 9 bp deletions, and some of these were specific and repeatedly recovered in independently-derived mice. There was no significant difference among the three injection methods. There is a risk of transgene integration even when they used the circular plasmids. Therefore, we performed PCR using a vector primers for DNA injected pups and find no integration of vector DNA (Supplementary Figure 2).

## Discussion

CRISPR/Cas is a rapid and efficient method for many species in which genome engineering has been difficult^11^,^12^,^13^,^14^,^15^,^16^,^17^,^18^. Recently, CRISPR/Cas engineering of the mouse genome using zygotes has been reported^7^. In this study, we evaluated the success rate and viability of three microinjection methods. We found that injection of RNA into the cytoplasm yields the best developmental efficiency at the blastocyst stage and in producing full-term mouse pups (Figure 2). This result suggests that the injection of DNA or RNA into the pronucleus is detrimental to the viability of the embryos. Injection of DNA could integrate into the chromosome and could have deleterious effects on embryonic development. However, we injected a circular plasmid DNA, which is difficult to integrate into chromosomes. In addition, the reason that injected RNA itself would have a deleterious effect is not clear. However, it is possible that the injection of gRNA into pronucleus could make more accessibility to targets and more damage to cells. Another explanation may be that the physical damage caused by injection of material into the pronucleus has an adverse effect on development. In the pronuclear stage zygotes, which were used for injection, chromosomes are expanded in the whole pronucleus. Therefore, the injection may damage the chromosomes. This could be the reason for the poor developmental efficiency of pronuclear injected embryos. Of course, a cytoplasmic injection could damage the cytoplasm; however, a pronuclear injection could damage both the cytoplasm and the pronucleus because the injection capillary necessarily moves through the cytoplasm before it reaches the pronucleus. The expression of protein amount and toxicity depends on the DNA/RNA concentration injected. Jaenisch's group reported that even embryos injected with 200 ng/μl of hCas9 RNA showed normal blastocyst development^8^. We injected with 50 ng/μl of hCas9 RNA and also showed the normal blastocyst development suggesting low toxicity.

The injection of RNA into the cytoplasm showed the best overall knockout efficiency normalized by the number of transferred zygotes. This result indicates that cytoplasmic injected guide RNAs can interact with the chromosomes and function as a guide to cleave chromosomal DNA. There are two possible mechanisms for this process. The guide RNAs may enter the pronucleus, or, alternatively, cleavage may occur after pronuclear envelope breakdown. If the latter is true, it could explain why the cytoplasmic RNA injection showed the best overall knockout efficiency. The total amount of RNA injected is larger in zygotes subjected to cytoplasmic injection because the volume of cytoplasm is larger than the nucleus. After pronuclear envelope breakdown, the pronucleus and cytoplasm are no longer separate and the amount of RNA injected determines the concentration of injected RNA. However, the percentage of pups produced by cytoplasmic injection (Figure 2b) and the overall percentage of knockout animals produced by cytoplasmic injection (Figure 4b) are similar, suggesting that the overall knockout efficiency of cytoplasmic injection reflects the level of viability.

The percentages of homozygous knockouts per KO mice were significantly lower in the mice that received an injection of DNA into the pronucleus, compared to the other two groups (Figure 4b). Injected DNA could express the RNA products for a longer time period compared to injected RNA. Therefore, the expression of Cas9 protein or guide RNA at levels high enough to generate homozygous deletions of the gene may have additional effects that are lethal for the embryos. However, this trend was not clearly seen in the KO mice made by a gRNA for exon 7 (Table 2).

Interestingly, most of the biallelic mutant mice have the same deletions (Figure 5). All of these mutations are 9 bp deletions, and some of these were specific and repeatedly recovered in independently-derived mice. Generation of these specific alleles is likely caused by a short sequence repeat flanking the cleavage sites. Previous reports demonstrated that perfect micro-homology sequences flanking the cleavage sites can generate micro-homology-mediated precise deletions via an end repair mechanism^19^,^20^ (Figure 5). TALEN mRNA injected one-cell rat embryos also showed similar deletions^21^.

## Methods

### Construction of gRNA-targeting vector for *Tet1*

Two oligonucleotides (Table 3) were annealed and extended to make a 100 bp double stranded DNA fragment including a target site and a vector using Phusion DNA polymerase (NEB). The gRNA cloning vector (http://www.addgene.org/41824/) was linearized using Afl II. The 100 bp DNA fragment was incorporated into the vector using Gibson assembly (NEB).

### Production of Cas9 mRNA and gRNA

A three nucleotide spacer (GCG) and the T7 promoter were added to the Cas9 coding region by PCR amplification using the Cas9 primers (Table 3) with pCAG-hCas9^22^ as the template. The amplified Cas9 PCR product was gel purified and used as the template for *in vitro* transcription (IVT) using mMESSAGE mMACHINE T7 ULTRA kit (Life Technologies). A four nucleotide spacer (GCGT) and the T7 promoter were added to the gRNA template plasmid by PCR amplification using the *Tet1* primers (Table 3). The amplified *Tet1* gRNA PCR product was gel purified and used as the template for IVT using a MEGAshortscript T7 kit (Life Technologies). Both the Cas9 mRNA and the gRNAs were purified using a MEGAclear kit (Life Technologies) and eluted into RNase-free water. The qualities of the RNAs were checked by gel electrophoresis.

### Animals

C57BL/6J mice were purchased from CLEA Japan. All animal experiments were approved by the Animal Care and Experimentation Committee of Gunma University and were carried out in accordance with the approved guidelines.

### Microinjection of zygotes

All animal procedures were approved by Animal Care and Experimentation Committee at Gunma University and the National Cerebral and Cardiovascular center, and carried out in accordance with approved guidelines. Female C57BL/6J mice (CLEA Japan) were superovulated by the injection of 7.5 units of pregnant mare's serum gonadotropin (PMSG; ASKA Pharmaceutical) followed by 7.5 units of human chorionic gonadotrophin (hCG; ASKA Pharmaceutical) 48 h later. Females were mated overnight with C57BL/6JJcl male mice. The following day, fertilized embryos were collected from the oviducts. For DNA injection, the Cas9 expression vector (2.5 ng/μl) and gRNA expression vector (2.5 ng/μl)^23^ were injected into the pronuclei of fertilized embryos in M2 medium. For mRNA injection, *in vitro* transcribed Cas9 mRNA (50 ng/μl) and gRNA (20 ng/μl)^8^ were injected into the pronuclei or cytoplasm of fertilized embryos. The Cas9 and gRNA injected embryos were cultured in KSOM with amino acids at 37°C under 5% CO_2_ in air. For the production of mutant mice, 1-cell stage embryos were transferred into the ampulla of the oviduct (9–18 embryos per oviduct) of pseudopregnant Jcl:ICR (CLEA Japan) females. To determine the developmental rate *in vitro*, embryos were cultured until the blastocyst stage (4.5 days).

### Assay for genome modification

To detect small genome modifications, PCR was performed using primers flanking the targeted region (Table 3). The PCR products were digested with Sac I, which cleaves at the Cas9 target site of the non-modified genomes. PCR products were then analyzed by agarose gel electrophoresis. Some of the PCR products were cloned into a TA-cloning vector (pCR2.1) and sequenced.

### Statistical analysis

Body weights of pups were analyzed with one-factor ANOVA, followed by Tukey-Kramer test. Percentages of blastocysts, pups and mutants were analyzed by chi-square test. A probability value of P < 0.05 was considered significant. All statistical analyses were performed using software (statcel2).

## Supplementary Material

Supplementary Information Supplementary Figures

## Figures and Tables

**Table 1:** *In vitro* development of CRISPR/Cas introduced embryos using three microinjection methods

gRNA	microinjection method	survived/injected	blastocyst/survived
	DNA/Pronucleus	41/52 (78.8%)	10/41 (24.4%)
Tet1 Ex4	RNA/Pronucleus	52/61 (85.2%)	17/52 (32.7%)
	RNA/Cytoplasm	46/63 (73.0%)	30/46 (65.2%)

DNAs or RNAs of hCas9 and gRNA were mixed and injected into mouse zygotes by three methods: (1) injection of DNA into the pronucleus, (2) injection of RNA into the pronucleus, and (3) injection of RNA into the cytoplasm.

**Table 2:** Efficiency of CRISPR/Cas-mediated gene targeting using three microinjection methods

gRNA	microinjection method	survived/injected	pups/transferred	KO/pups	homo/pups	wild:hetero:homo
	DNA/Pronucleus	62/82 (75.6%)	5/62 (8.1%)	4/5 (80.0%)	1/5 (20.0%)	1:3:1
Tet1 Ex4	RNA/Pronucleus	72/81 (88.9%)	5/72 (6.9%)	5/5 (100%)	5/5 (100%)	0:0:5
	RNA/Cytoplasm	55/72 (76.4%)	9/37 (24.3%)	9/9 (100%)	8/9 (88.9%)	0:1:8
	DNA/Pronucleus	41/54 (75.9%)	8/41 (19.5%)	2/8 (25.0%)	1/8 (12.5%)	6:1:1
Tet1 Ex7	RNA/Pronucleus	46/56 (82.1%)	15/46 (32.6%)	7/15 (46.7%)	3/15 (20.0%)	8:4:3
	RNA/Cytoplasm	31/46 (67.4%)	19/31 (61.3%)	10/19 (52.6%)	4/19 (21.1%)	9:6:4

DNAs or RNAs of hCas9 and gRNA were mixed and injected into mouse zygotes by three methods: (1) injection of DNA into the pronucleus, (2) injection of RNA into the pronucleus, and (3) injection of RNA into the cytoplasm. The injected eggs were transferred into pseudopregnant females. The mutations were identified by digestion of PCR amplified fragment containing target with a restriction enzyme at the hCas9 cleavage sites followed by agarose gel electrophoresis.

**Table 3:** Oligonucleotides used for construction of gRNA vector, genotyping, and IVT for Tet1Ex4. Those for Tet1Ex7 were previously reported^24^

Oligonucleotides for construction of gRNA vector	
Primer name	Sequence
Tet1Ex4gRNA-S1	TTTCTTGGCTTTATATATCTTGTGGAAAGGACGAAACACCGGCTGCTGTCAGGGAGCTCA
Tet1Ex4gRNA-AS1	GACTAGCCTTATTTTAACTTGCTATTTCTAGCTCTAAAACTGAGCTCCCTGACAGCAGCC

Primer for Genotyping	
Primer name	Sequence
Tet1Ex4-3	TTGTTCTCTCCTCTGACTGC
Tet1Ex4-4	TGATTGATCAAATAGGCCTGC

Primer for IVT	
Primer name	Sequence
IVTFd3 pCAG-hCas9	GCGTAATACGACTCACTATAGGGAGAATGGACAAGAAGTACTC
IVTRv pCAG-hCas9	AGAGTCGCGGCCGCTCAC
IVTgRNATet1Ex4RJ2	GCGTTAATACGACTCACTATAGGCTGCTGTCAGGGAGCTC
IVTgRNARv	AAAAGCACCGACTCGGTGCC
