# Supplementary material for: Validation of microinjection methods for generating knockout mice by CRISPR/Cas-mediated genome engineering
Source: Sci Rep. 2014 Mar 28;4:4513. doi: 10.1038/srep04513 (PMC5380110; doi:10.1038/srep04513)
Supplement: Supplementary Information — Supplementary Figures [file srep04513-s1.pdf]

Supplementary figures
of

Title: Validation of microinjection methods for generating knockout mice by CRISPR/Cas-mediated genome engineering

Authors: Takuro Horii[1, #], Yuji Arai[2, #], Miho Yamazaki[1,3,4], Sumiyo Morita[1], Mika Kimura[1], Masahiro Itoh[4], Yumiko Abe[3], Izuho Hatada[1, *]

Affiliations

1. Laboratory of Genome Science, Biosignal Genome Resource Center, Institute for Molecular and Cellular Regulation, Gunma University, 3-39-15 Showa-machi, Maebashi, Gunma 371-8512, Japan.
2. Division of Developmental Biotechnology, Department of Bioscience and Genetics Research Institute, National Cerebral and Cardiovascular Center, 5-7-1 Fujishiro-dai, Suita Osaka 565-8565, Japan.
3. Department of Laboratory Sciences, Graduate School of Health Sciences, Gunma University, 3-39-22 Showa-machi, Maebashi, Gunma 371-8514, Japan.
4. Department of Obstetrics and Gynecology, Gunma CHUO General Hospital, 1-7-13, Kouun-cho, Maebashi, Gunma 371-0025, Japan.

*Correspondence should be addressed to: hatada@gunma-u.ac.jp

[#] These authors contributed equally to this work.

Sup Figure 1. Body weight of *Tet1*Ex7 KO newborn mice

Body weight of newborn mice made by three methods were analyzed for genotype of *Tet1*.

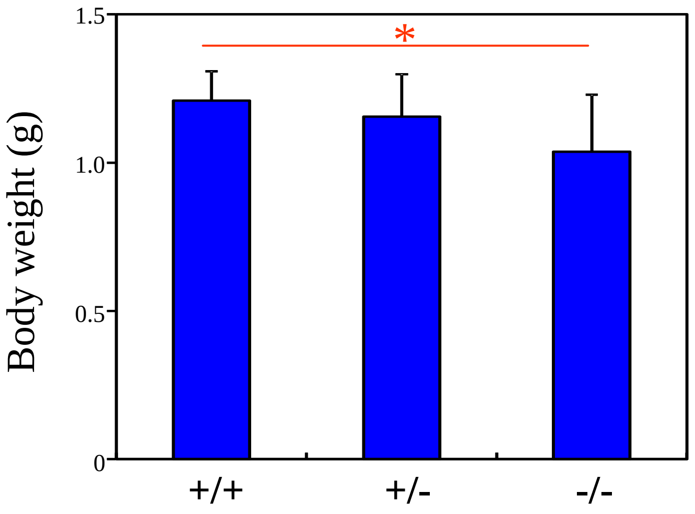

Sup Figure 2. pCAG-hCas9 vector PCR for (DNA injected mice)

94℃ 3min, 94℃ 10sec, 60℃ 30sec, 72℃ 1min, 72℃ 5min

x30

Template DNA: tail tips derived from pups (100ng/reaction)

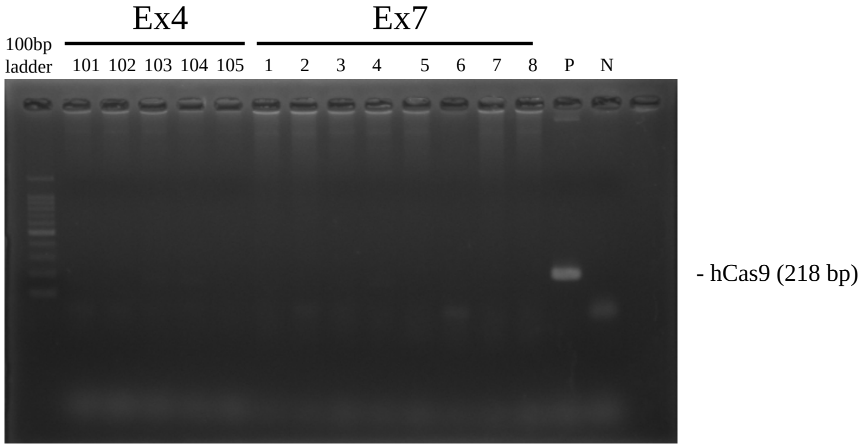

P: pCAG-hCas9 vector, N: water
